# Precautionary measures needed for ophthalmologists during pandemic of the coronavirus disease 2019 (COVID‐19)

**DOI:** 10.1111/aos.14438

**Published:** 2020-04-27

**Authors:** Kelvin H Wan, Suber S Huang, Alvin L Young, Dennis Shun Chiu Lam

**Affiliations:** ^1^ Department of Ophthalmology and Visual Sciences The Chinese University of Hong Kong Hong Kong; ^2^ Department of Ophthalmology Tuen Mun Hospital Hong Kong; ^3^ Retina Center of Ohio Cleveland Ohio USA; ^4^ Bascom Palmer Eye Institute University of Miami Miami Florida USA; ^5^ Department of Ophthalmology and Visual Sciences Prince of Wales Hospital Hong Kong; ^6^ C‐MER Dennis Lam & Partners Eye Center C‐MER International Eye Care Group Hong Kong; ^7^ Hong Kong International Eye Research Institute of the Chinese University of Hong Kong (Shenzhen) Shenzhen China

The novel coronavirus disease 2019 (COVID‐19) caused by severe acute respiratory syndrome coronavirus‐2 (SARS‐CoV‐2) emerged in December 2019 in Wuhan, China, has spread to over 113 countries with 118 326 infected and 4292 died as of 11 March 2020 and the World Health Organization (WHO) has just announced COVID‐19 a global pandemic. A person under investigation (PUI) for COVID‐19 is less likely to present initially to the ophthalmologists compared to emergency care or internal medicine physicians. However, in late February 2020, 2 patients presented simultaneously to our eye casualty with sudden onset of unilateral painful red eye associated with a decline in visual acuity; their intraocular pressure was over 40 mmHg, and slit‐lamp examination findings were suggestive of acute primary angle closure (APAC). The episodes aborted with topical treatment and laser iridotomy. A more elaborate history taking revealed they have been taking over‐the‐counter (OTC) cold/flu medication for respiratory symptoms and fever. Further enquiry about their history of travel identified a recent return from Mainland China. Given these patients fulfilling both the clinical feature and the epidemiological criteria for PUI proposed by the Centers for Disease Control and Prevention (CDC), they were admitted to the isolation ward and had nasopharyngeal aspirate and throat swab samples tested for SARS‐CoV‐2 and respiratory viruses. Both patients were negative for SARS‐CoV‐2 but were positive for respiratory syncytial virus and para‐influenza type 2 virus, respectively; they were discharged to the general medical ward subsequently. Over‐the‐counter (OTC) cold/flu medication can precipitate APAC in predisposed eyes such as those with an anatomically narrow angle. Although COVID‐19 and APAC are apparently unrelated, these two cases illustrate that ophthalmologists could be the first healthcare provider to evaluate suspected cases that present to us in inconspicuous ways.

Our specialty strongly relies on physical examination to make the diagnosis, which is performed at a short distance from the patient. The CDC defined close contact of being approximately 2 m from a patient for a prolonged duration, where any contact longer than 1–2 min of exposure is considered prolonged until more is known about transmission risks. The time it takes an ophthalmologist to complete a comprehensive ophthalmic examination is well beyond this duration. Despite we do not perform any aerosol‐generating procedures, the close proximity and prolonged duration of patient contact could increase our risk of exposure. A self‐made transparent polycarbonate protector mounted to the slit‐lamp offers a physical barrier between the patient and ophthalmologist while not interfering with its normal usage and patient interaction. Alternatives to direct ophthalmoscopy such as binocular indirect ophthalmoscope should be performed in view of the shorter working distance in the former.

The presence of SARS‐CoV‐2 in the tear film has been detected using real‐time reverse‐transcription–polymerase‐chain‐reaction (RT‐PCR) assays in the infected individuals (Xia et al. 2020). A medical expert who visited Wuhan developed conjunctivitis prior to the onset of respiratory symptoms; he was later tested positive to SARS‐CoV‐2, suggesting conjunctivitis could be one of the signs of COVID‐19 (Lu et al. 2020). We should, therefore, remain vigilant in attending a patient with conjunctivitis. In patients presenting with acute conjunctivitis but without any catarrhal symptoms or recent travel to affected geographic areas, conjunctival swab for RT‐PCR could be considered to address whether SARS‐CoV‐2 is found on the ocular surface and could also possibly aid in the earlier diagnosis in these subclinical cases if the facility is available and not too costly. Tear film disturbances have also been associated with non‐contact air‐puff tonometry, suggesting that this could be a micro‐aerosol formation procedure (Li et al. 2020). The intraocular pressure should be measured using alternative instruments as far as possible.

Substantial involvement of nosocomial transmission in both the SARS‐CoV outbreak in 2003 and the Middle East respiratory syndrome CoV outbreak in 2012 was evident. Given the similarity in genomic sequence between SAR‐CoV‐2 and these coronaviruses, the propensity for nosocomial spread for the current COVID‐19 should not be taken lightly, and measures should be taken to limit such transmission. Urgent consultations (penetrating ocular injury, acute glaucoma and alkali chemical injury, etc.) should be attended with adequate appropriate personal protection equipment (PPE), whereas non‐urgent cross‐specialty consultations for in‐patients should be referred to outpatient setting after discharge. For stable patients without changes in medications or drug‐related issues, prescription refill could reduce their trip to the clinic. Patients scheduled for elective surgery and laser treatment should be deferred in the midst of an outbreak. We should also ensure rapid triage and isolate suspected patients upon their arrival to the healthcare facility.

As much is still to be learned about COVID‐19, comparison with SARS‐CoV is often made and strategies adopted during the previous coronavirus pandemic could also be applied during the current outbreak (Chan et al. 2006). During the SARS‐CoV outbreaks, we carried out studies in Hong Kong to evaluate ophthalmic manifestation of SARS‐CoV by performing ocular screening, tear swabs and conjunctival scrapping in confirmed cases (Chan et al. 2006). No ophthalmologist involved in the care of these patients was infected; therefore, our standard of PPE used by the ophthalmologist during the SARS‐CoV pandemic could serve as a reference for PPE in the current coronavirus pandemic. Basically, the three‐pronged strategies are (1) protecting staff with appropriate PPE; (2) preventing spread of the virus from our patients; and (3) reengineering of workflow to minimize exposure time and/or risk of cross infections. We recommend following standard precaution and masks/respirators should be worn by everybody inside the ophthalmic practice. N95 respirators provide more protection but in case of shortage, surgical masks are good alternative for our day‐to‐day practice. However, full PPE including caps, gowns, N95 respirators and eye goggles for the protection of mouth, nose and eye should be worn in handling cases confirmed or PUI cases. Ensuring the safety of medical personnel is imperative to avoid spread of the virus but safeguard continuous patient care.

Recent evidence suggests that the SARS‐CoV‐2 could be transmitted via asymptomatic infected individuals. An asymptomatic index patient from Shanghai attended a meeting in Germany had no symptoms until her flight back to China. Two of this index patient's colleague who had close contact with her and another two colleagues who attended the meeting without close contact were later found to be infected with COVID‐19 (Rothe et al. 2020). Of the 114 asymptomatic predominantly German evacuated from Wuhan, who were labelled negative in a multistep process of signs and symptoms screening of infection, two were later tested positive for SARS‐CoV‐2 by RT‐PCR (Hoehl et al. 2020). Given the suboptimal effectiveness of symptom‐based screening process in detecting COVID‐19 in serologically positive cases and that transmission can occur during the incubation period in asymptomatic individuals, this highlight the importance of our proposed precautionary measures as the transmission dynamics, infective potency and epidemiology are changing on a daily basis during the ongoing COVID‐19 pandemic. Clinics and hospitals are places that people do not want to go during a pandemic. It can be envisaged that interaction between doctors and patients through the Internet with the aid of artificial intelligence will become more and more important (Balyen & Peto 2019; Tan et al. 2019).

## References

[aos14438-bib-0001] Balyen L & Peto T (2019): Promising artificial intelligence‐machine learning‐deep learning algorithms in ophthalmology. Asia Pac J Ophthalmol (Phila) 8: 264–272. 31149787 10.22608/APO.2018479

[aos14438-bib-0002] Chan WM , Liu DT , Chan PK et al. (2006): Precautions in ophthalmic practice in a hospital with a major acute SARS outbreak: an experience from Hong Kong. Eye (Lond) 20: 283–289. 15877099 10.1038/sj.eye.6701885 PMC7091695

[aos14438-bib-0003] Hoehl S , Rabenau H , Berger A et al. (2020): Evidence of SARS‐CoV‐2 infection in returning travelers from Wuhan, China. N Engl J Med 382: 1278–1280. 32069388 10.1056/NEJMc2001899 PMC7121749

[aos14438-bib-0004] Lu CW , Liu XF & Jia ZF (2020): 2019‐nCoV transmission through the ocular surface must not be ignored. Lancet 395: e39. 32035510 10.1016/S0140-6736(20)30313-5 PMC7133551

[aos14438-bib-0005] Li C , Tang Y , Chen Z , Wang A , Huang X , Chen Y & Qu J (2020): Aerosol formation during non‐contact 'air‐puff' tonometry and its significance for prevention of COVID‐19. Chin J Exp Ophthalmo 38: 212–216.

[aos14438-bib-0006] Rothe C , Schunk M , Sothmann P et al. (2020): Transmission of 2019‐nCoV infection from an asymptomatic contact in Germany. N Engl J Med 382: 970–971. 32003551 10.1056/NEJMc2001468 PMC7120970

[aos14438-bib-0007] Tan Z , Scheetz J & He M (2019): Artificial intelligence in ophthalmology: accuracy, challenges, and clinical application. Asia Pac J Ophthalmol (Phila) 8: 197–199. 31179666 10.22608/APO.2019122

[aos14438-bib-0008] Xia J , Tong J , Liu M , Shen Y & Guo D (2020): Evaluation of coronavirus in tears and conjunctival secretions of patients with SARS‐CoV‐2 infection. J Med Virol. 10.1002/jmv.25725. In press. PMC7228294 32100876

